# Strategy for mass production of lytic *Staphylococcus aureus* bacteriophage pSa-3: contribution of multiplicity of infection and response surface methodology

**DOI:** 10.1186/s12934-021-01549-8

**Published:** 2021-03-02

**Authors:** Sang Guen Kim, Jun Kwon, Sib Sankar Giri, Saekil Yun, Hyoun Joong Kim, Sang Wha Kim, Jung Woo Kang, Sung Bin Lee, Won Joon Jung, Se Chang Park

**Affiliations:** Laboratory of Aquatic Biomedicine, College of Veterinary Medicine and Research Institute for Veterinary Science, Seoul National University, Seoul, 08826 Republic of Korea

**Keywords:** Lytic bacteriophage, *Staphylococcus* phage, Mass production, Optimization, Multiplicity of infection, Response surface methodology

## Abstract

**Background:**

Antibiotic-resistant bacteria have emerged as a serious problem; bacteriophages have, therefore, been proposed as a therapeutic alternative to antibiotics. Several authorities, such as pharmacopeia, FDA, have confirmed their safety, and some bacteriophages are commercially available worldwide. The demand for bacteriophages is expected to increase exponentially in the future; hence, there is an urgent need to mass-produce bacteriophages economically. Unlike the replication of non-lytic bacteriophages, lytic bacteriophages are replicated by lysing host bacteria, which leads to the termination of phage production; hence, strategies that can prolong the lysis of host bacteria in bacteria–bacteriophage co-cultures, are required.

**Results:**

In the current study, we manipulated the inoculum concentrations of *Staphylococcus aureus* and phage pSa-3 (multiplicity of infection, MOI), and their energy sources to delay the bactericidal effect while optimizing phage production. We examined an increasing range of bacterial inoculum concentration (2 × 10^8^ to 2 × 10^9^ CFU/mL) to decrease the lag phase, in combination with a decreasing range of phage inoculum (from MOI 0.01 to 0.00000001) to delay the lysis of the host. Bacterial concentration of 2 × 10^8^ CFU/mL and phage MOI of 0.0001 showed the maximum final phage production rate (1.68 × 10^10^ plaque forming unit (PFU)/mL). With this combination of phage–bacteria inoculum, we selected glycerol, glycine, and calcium as carbon, nitrogen, and divalent ion sources, respectively, for phage production. After optimization using response surface methodology, the final concentration of the lytic *Staphylococcus* phage was 8.63 × 10^10^ ± 9.71 × 10^9^ PFU/mL (5.13-fold increase).

**Conclusions:**

Therefore, *Staphylococcus* phage pSa-3 production can be maximized by increasing the bacterial inoculum and reducing the seeding phage MOI, and this combinatorial strategy could decrease the phage production time. Further, we suggest that response surface methodology has the potential for optimizing the mass production of lytic bacteriophages.

**Supplementary Information:**

The online version contains supplementary material available at 10.1186/s12934-021-01549-8.

## Background

Antibiotic-resistant bacteria, such as vancomycin-resistant Enterococci, methicillin-resistant *Staphylococcus aureus*, and carbapenem-resistant *Enterobacteriaceae*, are a global, emerging problem [1–3]. With the emergence of these antibiotic-resistant bacteria, an alternative treatment strategy is urgently required. One of the most anticipated alternatives is the use of lytic bacteriophages (phages), which are effective against bacterial infections [4–6]. Numerous phages are commercially available, and their therapeutic application is reported from East European countries, such as Georgia, Russia, Poland, and Belgium [7]; several phages have also been approved by the US FDA, as they have been proven to have no adverse effects during phase 1 clinical trials [8, 9]; hence, phage therapy has attracted attention as a potential alternative to antibiotics.

The most important aspect in phage therapy is the isolation and selection of effective phages against antibiotic-resistant bacteria [10–12]. Mass production of these phages is the next pivotal step based on increasing demands in the future [13–16]. It is well known that host bacterial cells and their phages have different optimal conditions for replication. As described in previous reports, replication of phages is dependent on the physiology of their hosts [17, 18]. In particular, the specific growth rate (μ) of host bacteria is an important factor for the replication of phages [19, 20].

Temperate or filamentous phages replicate in proportion to the growth of the host, without a significant effect on the host [21, 22]. In contrast, the replication of lytic phages causes lysis of their host, which means there will be no “factory” to produce phages in the end. Thus, keeping host bacteria alive for longer periods by manipulating the physiology or the interaction between phage and bacteria can be a strategy for increasing the yield of lytic phages [23]. In addition to manipulating the nature of bacteria, scale-up trials (i.e. flask to fermenter) are being widely researched [24, 25].

Previously, we isolated the obligatory lytic phage pSa-3 which effectively lysed *S. aureus* cells at low concentration (MOI 0.1) [26, 27]. The phage pSa-3 which has 137,836 base pair (bp) of genomic DNA was classified in the genus *Kayvirus*, family *Herelleviridae.* With the co-application of a surfactant, the *Staphylococcus* phage pSa-3 could effectively degrade the aggregations of *S. aureus* including biofilms in vitro and in vivo [26]. As this phages does not possess any antibiotic resistance- or virulence-related genes, or lysogeny-related genes, pSa-3 has the potential to be used as a therapeutic agent for the treatment of *S. aureus*-associated atopic dermatitis [27].

This study was designed to optimize the phage yield by altering the physiology of bacteria by controlling the energy source and by manipulating the bacterial inoculum and phage concentration to keep the host alive for a longer period. Carbon, nitrogen, ion sources, and surfactant were supplemented in the growth medium, and the multiplicity of infection (MOI) was modified for the maximization of the phage production yield. Response surface methodology (RSM) was also adopted for the optimization of the combined effect.

## Results

### Effect of MOI on phage amplification

**Fig. 1:**
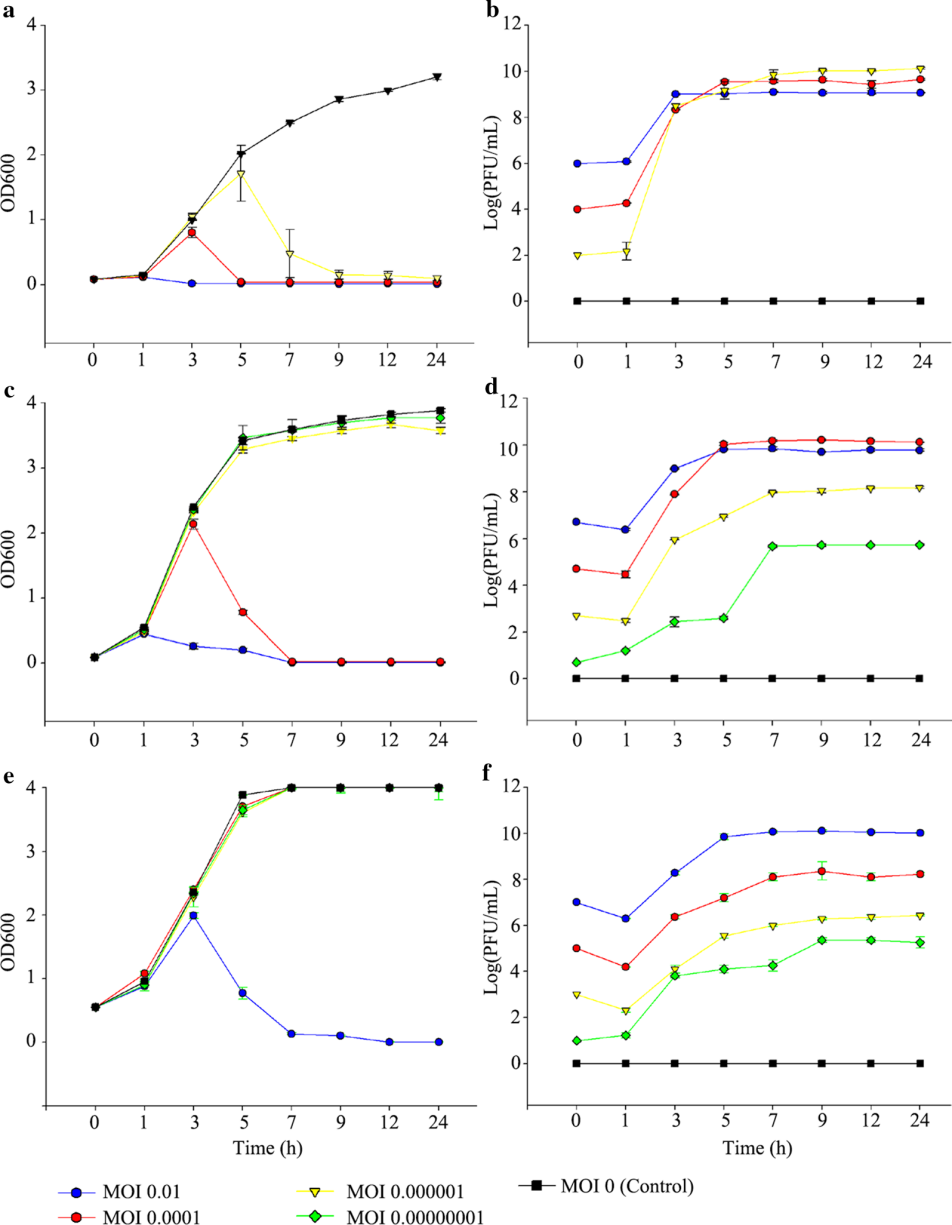
Growth curve of the microorganisms in the bacteria–phage co-culture. Bacterial growth was measured using optical density (**a**, **c**, **e**). Phage growth was measured in PFU count (**b**, **d**, **f**). 2 × 10^8^ CFU/mL of initial *Staphylococcus aureus* concentration (**a**, **b**), 1 × 10^9^ CFU/mL of *S. aureus* concentration (**c**, **d**), and 2 × 10^9^ CFU/mL of *S. aureus* concentration (**e**, **f**). The experiment was performed in triplicate

The effect of MOI on phage amplification is shown in Fig. 1. The experiment was conducted with various bacterial inoculum concentrations to analyze the effect of increased bacterial load on phage production yield. As shown in Fig. 1a, the host bacteria were lysed 9 h after phage injection in all groups. In the 1% bacterial inoculum group (initial concentration: 2 × 10^8^ CFU/mL), the phage yield was negatively correlated with the phage inoculum, whereas the final phage yield was positively correlated with the bacterial optical density (OD; Fig. 1b). In the 5% bacterial inoculum group (initial concentration: 1 × 10^8^ CFU/mL), the lower MOI groups (MOI 0.00000001 and 0.000001) showed bacterial growth, while the other groups showed inhibition of bacterial growth (Fig. 1c). As shown in Fig. 1d, MOI 0.01 and 0.0001 groups showed the highest phage yield (approximately 10^10^ PFU/mL), while the lowest MOI group (MOI 0.000001) showed lower phage production (approximately 10^8^ PFU/mL) than the other groups. In the 10% bacterial inoculum group (initial concentration: 2 × 10^9^ CFU/mL), the two lower MOI groups (MOI 0.000001 and 0.00000001) showed bacterial growth rather than replication of phages similar to the 5% bacterial inoculum–MOI 0.000001 group (Fig. 1e), and only the MOI 0.01 group showed lysis of the host bacteria. The MOI 0.01 group showed the highest phage production (approximately 10^10^ PFU/mL), while the lower MOI groups produced phages fewer than 10^9^ PFU/mL (Fig. 1f). As the MOI (phage inoculum) was lowered, an increase in the time at which the inhibition of bacterial growth began was observed, and the final concentration of phages did not increase after reaching the highest concentration for 24 h. The summary of the maximum phage production yield in each bacterial inoculum group is presented in Table 1. The group with 5% bacterial inoculum–MOI 0.0001, which showed the highest phage yield, was used for further experiments.

**Table 1:** Effects of MOI on lytic *Staphylococcus aureus* phage pSa-3 production

Bacterial inoculum (CFU/mL)	Phage inoculum (MOI)	Production_max_^a^ ($\times$ 1^10^ PFU/mL)	Production time^b^ (h)
2 × 10^8^	0.01	(1.27 ± 0.057) × 10^–1^	7
	0.0001	(4.27 ± 0.551) × 10^–1^	9
	0.000001	(1.36 ± 0.112) × 10^0^	24
1 × 10^9^	0.01	(7.27 ± 1.36) × 10^–1^	7
	0.0001	(1.68 ± 0.176) × 10^0^	9
	0.000001	(1.54 ± 0.175) × 10^–2^	24
	0.00000001	(5.57 ± 0.17) × 10^–5^	24
2 × 10^9^	0.01	(1.25 ± 0.15) × 10^0^	9
	0.0001	(3.00 ± 2.65) × 10^–2^	9
	0.000001	(2.73 ± 0.17) × 10^–4^	24
	0.00000001	(2.00 ± 1.00) × 10^–5^	24

^a^The phage concentration produced to the maximum. The values were shown as mean ± SD from triple replicates

^b^Time to produce the maximum phage concentration

### Effect of growth medium source on phage amplification

**Fig. 2:**
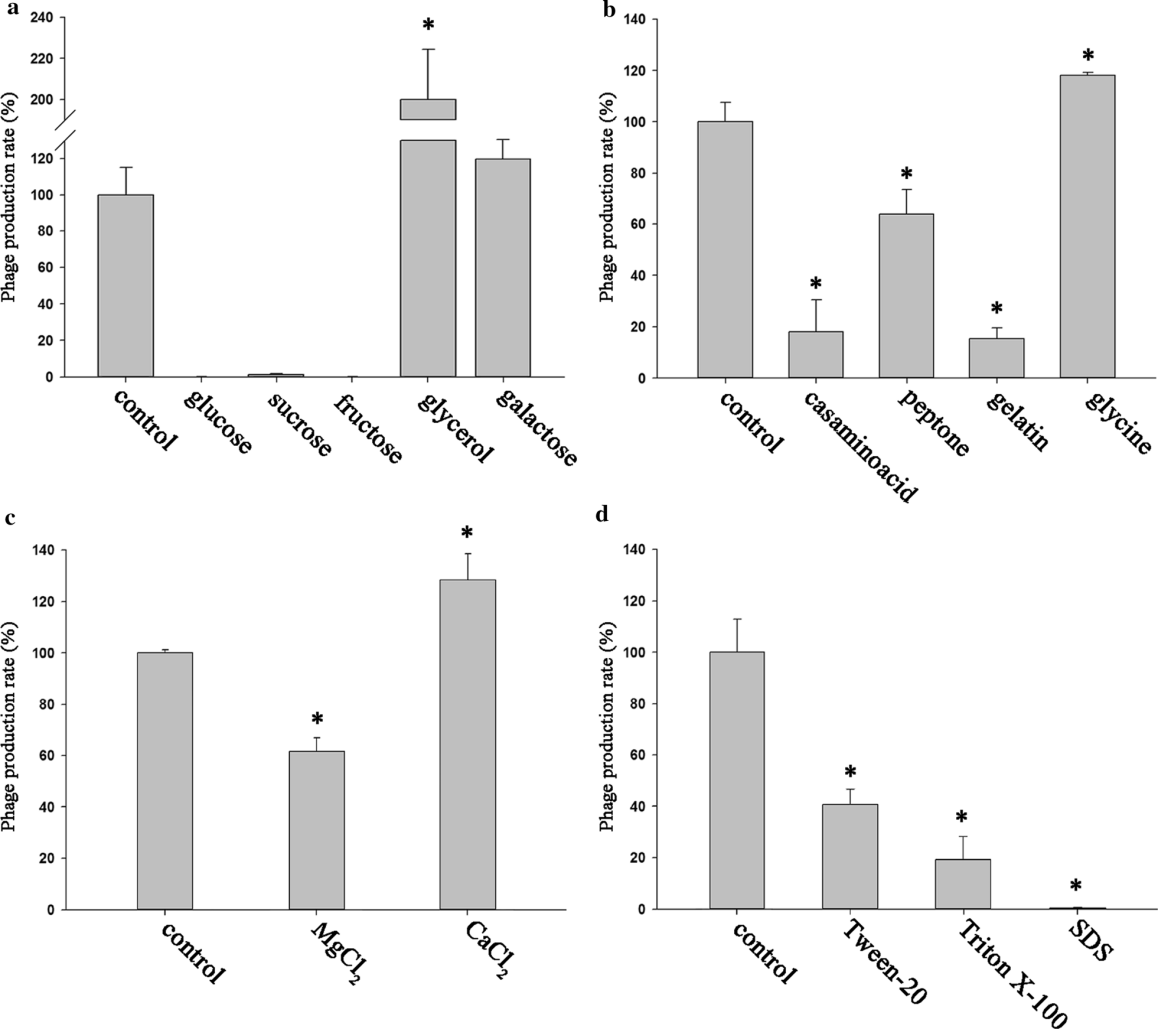
Media supplement screening for maximizing the lytic *Staphylococcus aureus* phage pSa-3 production with carbon sources (**a**), nitrogen sources (**b**), divalent ion sources (**c**), and surfactants (**d**). The experiment was performed in triplicate. Statistical significance was calculated using Student’s t-test, and p < 0.05 was considered as statistically significant

The effect of medium supplements on bacteriophage production was analyzed using 5% bacterial inoculum with MOI of the phage being 0.0001, as the combination resulted in maximum production. As a medium supplement, different carbon sources, nitrogen sources, ions, and surfactants were supplemented in Luria–Bertani (LB) broth and analyzed using a one-factor-at-a-time method. As a carbon source, 0.5% (w/v) of glucose, sucrose, fructose, galactose, or glycerol was supplemented. Galactose and glycerol showed an upregulation of phage production, and glycerol supplementation led to a significant increase (p < 0.05), i.e., 100% increased value in phage production compared to that of the control (Fig. 2a). As a nitrogen source, 0.1% (w/v) of casamino acid, peptone, gelatin, or glycine was supplemented. Among the nitrogen sources, only glycine showed 18% increase in phage yield and was selected as a nitrogen source for further experiments (Fig. 2b). A divalent ion source, such as magnesium chloride or calcium chloride, was supplemented in the culture medium for the amplification test, and calcium chloride was selected as the ion supplement, as it revealed the highest (28% increase) yield (Fig. 2c). For the surfactant screening, tween 20, triton X-100, and SDS were examined, and these were found to downregulate phage amplification (Fig. 2d). Thus, glycerol, glycine, and calcium chloride were selected as carbon, nitrogen, and ion sources, respectively, for RSM analysis. And each supplement influenced the growth of the host bacteria, as well (Additional file 1: Fig. S1). Especially, SDS showed the deteriorating effect on host growth.

### Optimization of bacteriophage pSa-3 production by RSM

**Fig. 3:**
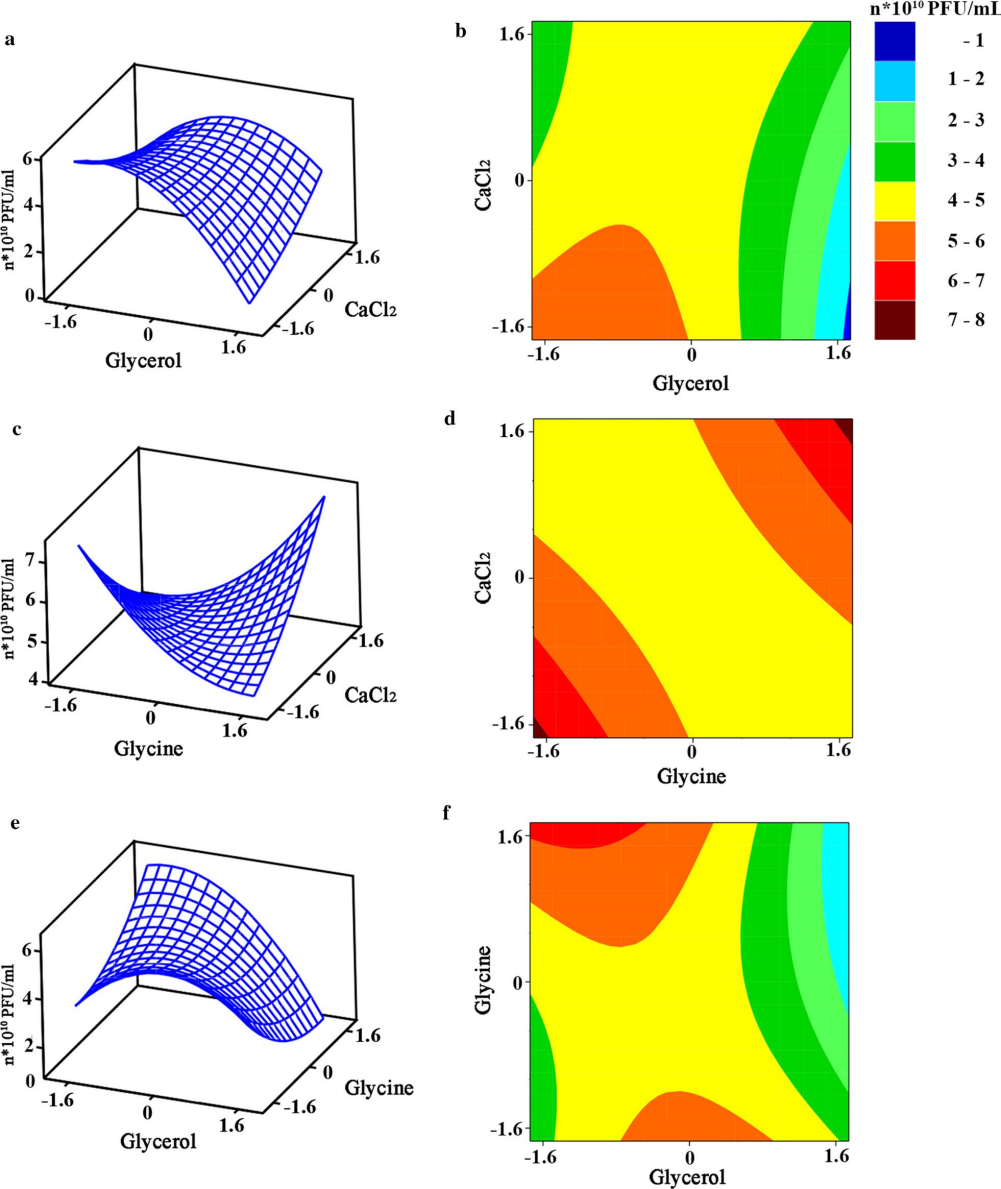
Response surface contour plots of media supplements for lytic *Staphylococcus aureus* phage pSa-3 production in 3D (**a**, **c**, **e**) and 2D (**b**, **d**, **f**). The effect of glycerol and calcium chloride on phage production (**a**, **b**). The effect of glycine and calcium chloride on phage production (**c**, **d**). The effect of glycerol and glycine on phage production (**e**, **f**)

RSM based on central composite design (CCD) technique was used to examine the effect of the selected media supplements on the amplification of the lytic phage pSa-3. A total of 20 coded levels are represented in Table 2. The experimental design and results obtained from RSM are shown in Table 3. By employing analysis of variance (ANOVA), the following equation was obtained, describing the relationship between phage yield (Y), and glycerol (X_1_), glycine (X_2_), and calcium chloride (X_3_):

$$\begin{aligned} {\text{Y}} & = 46287782214 - 7470787473{\text{X}}_{1} + 89924699{\text{X}}_{2} - 125989578{\text{X}}_{3} - 6578793992{\text{X}}_{1}^{2} + 2964876080{\text{X}}_{2}^{2} \\ & \quad + 1314889898{\text{X}}_{3}^{2} - 5212500000{\text{X}}_{1} {\text{X}}_{2} + 4745833333{\text{X}}_{1} {\text{X}}_{3} + 5879166667{\text{X}}_{2} {\text{X}}_{3} , \\ \end{aligned}$$

where Y represents the predicted phage yield; X_1_, X_2_, and X_3_ are the concentrations of supplemented glycerol, glycine, and calcium chloride, respectively. The experimental value varied from 2.00 × 10^10^ to 6.30 × 10^10^ PFU/mL (119% to 375% yield increase compared to that of the yield of MOI optimization), which fits in with the predicted value calculated from the equation. Figure 3 graphically represents the equation in 2D contour (Fig. 3a, c, e) and 3D response surface plot (Fig. 3b, d, f), which suggests the optimum range of the factors for the maximum phage yield response. The optimum conditions for increased phage yield are as follows: (a) glycerol concentration: − 1.3 to − 0.8, (b) glycine concentration at both ends: − 1.633 to 0 and 0 to 1.633, and (c) decrease of calcium chloride concentration from 1.633 to − 1.633.

**Table 2:** The Central Composite Design (CCD) of experiments and response of *Staphylococcus aureus* phage pSa-3 production

Standard order	Variables (coded value)			Variables (experimental value)		
	Glycerol *X*_1_	Glycine *X*_2_	CaCl_2_ *X*_3_	Glycerol (mg)	Glycine (mg)	CaCl_2_ (mM)
1	− 1	− 1	1	250.0	25.0	15.0
2	1	− 1	− 1	750.0	25.0	5.0
3	1	1	1	750.0	75.0	15.0
4	− 1	1	− 1	250.0	75.0	5.0
5	0	0	0	500.0	50.0	10.0
6	0	0	0	500.0	50.0	10.0
7	1	1	− 1	750.0	75.0	5.0
8	0	0	0	500.0	50.0	10.0
9	− 1	− 1	− 1	250.0	25.0	5.0
10	1	− 1	1	750.0	25.0	15.0
11	− 1	1	1	250.0	75.0	15.0
12	0	0	0	500.0	50.0	10.0
13	1.633	0	0	908.0	50.0	10.0
14	0	0	− 1.633	500.0	50.0	1.8
15	0	0	1.633	500.0	50.0	18.1
16	0	− 1.633	0	500.0	9.0	10.0
17	0	1.633	0	500.0	90.0	10.0
18	0	0	0	500.0	50.0	10.0
19	0	0	0	500.0	50.0	10.0
20	− 1.633	0	0	91.0	50.0	10.0

**Table 3:** The experimental design and response of lytic *Staphylococcus aureus* phage pSa-3 production

Standard Order	Glycerol *X*_1_	Glycine *X*_2_	CaCl2 *X*_3_	Observed values ($\times$10^10^ PFU/mL)	Predicted values ($\times$10^10^ PFU/mL)
1	− 1	− 1	1	1.74 ± 1.42	4.81
2	1	− 1	− 1	4.54 ± 0.17	3.78
3	1	1	1	3.57 ± 0.29	3.78
4	− 1	1	− 1	8.31 ± 0.93	4.81
5	0	0	0	5.11 ± 0.41	4.36
6	0	0	0	5.12 ± 0.23	4.36
7	1	1	− 1	2.01 ± 0.21	3.77
8	0	0	0	5.09 ± 0.18	4.36
9	− 1	− 1	− 1	9.41 ± 2.08	4.81
10	1	− 1	1	2.88 ± 0.40	3.79
11	− 1	1	1	4.05 ± 1.85	4.81
12	0	0	0	5.06 ± 0.28	4.36
13	1.633	0	0	1.98 ± 0.42	3.35
14	0	0	− 1.633	5.26 ± 0.26	4.36
15	0	0	1.633	6.83 ± 0.69	4.36
16	0	− 1.633	0	7.07 ± 1.10	4.36
17	0	1.633	0	7.60 ± 1.24	4.35
18	0	0	0	5.20 ± 0.26	4.36
19	0	0	0	5.13 ± 0.31	4.36
20	− 1.633	0	0	4.05 ± 0.75	5.03

### Analysis of variance

To validate the models used in this study, ANOVA was performed for the phage production yield. The significance of the models was determined by p-value less than 0.05. A low p-value of 0.023 with a high F value of 4.51 implied that the model was credible (Table 4). The analysis represented that the determination coefficient (R^2^) value of 0.84 and a p-value for lack of fit (0.084) were higher than the significance value (0.05). Overall, these statistical values suggested that RSM can be an effective tool for the optimization of lytic phage production, indicating that the model for the phage pSa-3 production in this study was a good fit.

**Table 4:** Analysis of variance (ANOVA) of the experimental results for the lytic *Staphylococcus aureus* phage pSa-3 production

	DF	Adj sum of squares	Adj mean square	F value	P value
Regression	9	6.53243E+21	8.96997E+19	0.47	0.639
*X*_1_	1	1.44869E+21	1.44869E+21	7.65	0.024
*X*_2_	1	4.30955E+17	4.30955E+17	0.00	0.963
*X*_3_	1	6.74732E+30	6.74732E+20	3.56	0.096
*X*_1_^2^	1	1.48521E+21	1.33629E+21	7.06	0.029
*X*_2_^2^	1	4.84644E+20	4.96999E+20	2.62	0.144
*X*_3_^2^	1	2.20061E+19	2.20061E+19	0.12	0.742
*X*_1_*X*_2_	1	1.16790E+20	1.16790E+20	0.62	0.455
*X*_1_*X*_3_	1	1.74936E+21	1.74936E+21	9.24	0.016
*X*_2_*X*_3_	1	5.50567E+20	5.50567E+20	2.91	0.127
Residual error	8	1.51494E+21	1.89367E+20		
Lack of fit	5	1.51462E+21	3.02925E+20	2890.10	0.000
Pure error	3	3.14444E+17	1.04815E+17		
Total	19	8.22677E+21			

### Validation of the optimized conditions

Based on the RSM analysis, the optimal phage yield was predicted to be 7.63 × 10^10^ PFU/mL when the code levels of glycerol, glycine, and calcium chloride were − 0.610313 (around 347 mg/mL of glycerol), 1.633 (90 mg/mL of glycine), and 1.633 (18 mM of calcium chloride), respectively. The validation experiment was conducted to confirm the predicted result. The observed average phage yield under the optimal value was 8.63 × 10^10^ ± 9.71 × 10^9^ PFU/mL, which was in good agreement with the predicted value.

## Discussion

The emergence of super-bacteria with resistance to multiple commonly used antibiotics has drawn the attention of scientists to bacteriophages as an alternative for antibiotics [28–30]. For therapeutic purposes, rapid eradication of pathogenic bacteria is important. Therefore, phage therapy research focused on isolating and selecting phages that can effectively control multidrug-resistant bacteria is gaining popularity [10–12, 31, 32]. On the contrary, for phage mass production, host bacteria are essential and should survive longer as “phage-producing factories.” In this study, the modification of the initial inoculum concentrations of the host and lytic phage improved the production rate of the phages. Meanwhile, the selected supplements successfully enhanced the lytic phage production after optimization by response surface methodology at bench scale (flask).

One approach to slowly lyse the “factory,” the host bacteria, is to lower the inoculum of phages pSa-3. In this study, we focused on the fact that *S. aureus* is gradually eradicated or partially suppressed at a low MOI of pSa-3, as described in the in vitro phage therapy studies [11, 33–36]. To verify that a lower phage inoculum results in higher phage production, we designed the study as follows [37, 38]. We used MOI 0.01 as the highest phage inoculum for the experiments, as MOI 0.1 was sufficient for the eradication of the host bacteria in our previous study [26]. In the first test with 2 × 10^8^ CFU/mL of bacteria and MOI 0.01 to 0.000001 of the phage, the lowest MOI resulted in the highest final phage production and delayed eradication of the host bacteria (Fig. 1). As the inoculum size and lag phase has a negative correlation in general, we expanded the initial bacterial concentration to 2 × 10^9^ CFU/mL for the reduction in production time and validation of the hypothesis “lower phage inoculum is good for production.” We observed that there might be a negative correlation between the initial phage inoculum and the final phage production or the phage production time; however, this was not always the case (Fig. 1). Although more detailed kinetics between the inoculum quantity of phage and bacteria should be elucidated through further studies, we observed that there was a lower limit of MOI (i.e., MOI 0.0001 and 0.01 for 1 × 10^9^ CFU/mL and 2 × 10^9^ CFU/mL initial concentration, respectively) for the final phage production. Simple manipulation of the initial phage and bacterial inoculum could regulate the production time and the final yield of the phage, without any other complicated alterations in culture conditions.

The optimal environments for the growth of phages and bacteria can be different; however, phages can control their life cycle in response to the physiology of the host [17–20, 39]. We examined the amplification rate by manipulating the inoculum dose and medium substrates such as carbon and nitrogen sources, divalent cations, and surfactants.

The carbon source can affect phage multiplication by physically modifying the host cell, such as by altering the receptors required for phage infection. Depending on the carbon supplement, the host cell surface area, which is positively correlated with phage adsorption and composition of receptors for phage infection, can be modified [17]. Previously, glycerol enhanced the size of plaques, indicating an increased number of the phage progeny in the multiplication process (Fig. 2a) [40].

While carbon supplement influences the physical factors involved in adsorption, the nitrogen source is also a major contributor to phage multiplication from a physiological perspective. A lack of a proper nitrogen source prevents the propagation of phages, whereas the carbon source does not [41]. Accordingly, numerous nitrogen sources such as amino acids, peptides, and proteins were used as supplemental nitrogen sources; a very small amount of these supplements was sufficient to increase the adsorption rate or the number of the phage progeny: glycine (13 mM) increased propagation (4.2-fold); tryptophan (1 g/L) increased adsorption (1.8-fold); peptone (0.4 g/L) increased propagation (100-fold); tryptone (1%) increased adsorption and propagation (two- to threefold); and gelatin (0.5 g/L) increased propagation (four- to sixfold) [42–46]. Positive results were observed only for glycine, as numerous differences including the microorganisms, culture media, or supplementation concentration were affected (Fig. 2b). Particularly, modifications with glycine supplement can upregulate the phage production because glycine showed synergistic effects with various materials (inorganic salts, purines, carbon dicarboxylic acids, and phosphorylated compounds) [47].

Metal ions such as divalent cations are well-known to affect phage multiplication [46]. Particularly, Ca^2+^ and Mg^2+^ examined in the present study are associated with the adsorption, penetration, and growth of virion progeny in host cells [48, 49]. Calcium is generally considered as a more dominant factor than magnesium; the burst size of the Ca^2+^-supplemented group was superior to that of the Mg^2+^-supplemented group (Fig. 2c) [50]. To exert the same effect on the burst size, much more Mg^2+^ is required [46]. Further, a reduced burst size was observed in some phages after supplementation with Mg^2+^, as shown in our result (Fig. 2c) [51]. In some cases, combined use of Ca^2+^ and Mg^2+^ had synergistic effects on the burst size [50]. Thus, combinations of divalent cations should be tested after RSM optimization to upregulate phage production.

*Staphylococcus aureus* cells aggregate into a grape pattern and form micro-colonies, decreasing the surface area of the individual cells. As described for the carbon source, a larger host surface area has greater chance of enabling a collision between a phage and its host [17]. Thus, it is necessary to reduce the adhesive form to increase phage multiplication. As surfactants possess anti-adhesive potential, they may be useful as therapeutic agents for *S. aureus* [52]. In our previous research, we observed that the use of a surfactant effectively reduced the size of the *S. aureus* micro-colonies [27]. Thus, we tested several surfactants, and found that they were not effective for phage multiplication (Fig. 2d). The mechanisms of phage multiplication facilitated by the surfactant should be further evaluated except the SDS which negatively influenced the host growth (Additional file 1: Fig. S1).

Each of the above-mentioned optimal parameters (except for the surfactants), when applied in combination, increased the final phage production, eventually showing a high production of 10.93 ± 0.9 log_10_PFU/mL (500% increase). However, the production of large phages from the *Herelleviridae* family is not as easy as the production of small phages of the *Podoviridae* and *Siphoviridae* families, which show a large propagation potential (burst size > 100 PFU/cell) [26, 53–55]. As they can propagate with small burst sizes, K (60 PFU/cell), pSa-3 (40 PFU/cell, this study), and phiPLA-RODI (25 PFU/cell), philPLA-RODI were propagated to a low concentration of 8.9 ± 0.1 log_10_PFU/mL using conventional methods [56–58]. Similarly, pSa-3 showed a low yield of approximately 5–6 log_10_PFU/mL before MOI optimization (Fig. 1). After RSM modification, philPLA-RODI and pSa-3 reached high concentrations (9.25 ± 0.9, and 10.93 ± 0.9 log_10_PFU/mL), with around 270% and 500% increase in yield. Although the two studies with the two phages used different factors for optimization, RSM analysis was reliable for the mass production of phages. Thus, a combined RSM experimental design using various factors may further increase the yield. In future, the demand for phages is expected to increase rapidly; therefore, economical mass production of phages is essential, and studies should focus on the optimization of phage mass production.

## Conclusions

This study aimed to develop an economical method for the mass production of lytic bacteriophages. We found that the manipulation of the initial inoculation ratio of the phage and its host can significantly influence the phage pSa-3 yield. However, the common belief that low MOIs guarantee high yields is untrue: there exists a lower limit of the inoculum ratio between the host and phage. After screening several substrates using the one-factor-at-a-time method, we were able to achieve more than 500% increase in the phage yield using RSM. Although we could not elucidate a correlation between the results from previous studies and those of the current study, we established that the manipulation of the bacterial inoculum and MOI will help in ensuring economically efficient mass production of phages in the future.

## Methods

### Microbial culture, growth conditions, and phage preparation

*Staphylococcus aureus* ATCC25923 and previously isolated phage pSa-3 were used for the experiments in this study [26, 27]. LB broth and agar media were used for the culture of the host bacteria and bacteriophages pSa-3. For phage preparation, 1% (2 × 10^8^ CFU) of overnight (approximately 18 h) bacterial culture adjusted to 2 × 10^8^ CFU and serial dilutions of the phage solution (10^2^–10^7^ PFU/mL) were co-inoculated in fresh LB broth and incubated overnight at 37 °C under shaking conditions (150 rpm). The culture was centrifuged (14,000×*g*), filtered (0.45 μm), and purified using the PEG precipitation method [59]. A high concentration (approximately 10^10^ PFU/mL) of the phage solution was stored at 4 °C for further use.

### Growth of bacteria and enumeration of bacteriophages

Bacterial growth and the concentration of the phages were examined periodically (0, 1, 3, 5, 7, 9, 12, and 24 h). For bacterial growth, OD was measured at 600 nm using a spectrophotometer (Biorad SmartSpecTM Plus, USA), and the number of phages was determined using a standard double layer agar plaque assay. Briefly, 100 μL of each of the diluted samples and the overnight bacterial culture were inoculated in the top agar (0.4% agar) and poured onto the bottom agar (1.5% agar) plate. After overnight incubation at 37 °C, the number of plaques was counted.

### Effect of MOI on phage amplification

The effect of MOI on phage amplification was examined with various combinations of bacterial inoculum and phage MOI after maintaining the bacteria–bacteriophage co-culture for 24 h. Briefly, 100 mL of fresh LB broth was inoculated with an overnight bacterial culture (adjusted to 2 × 10^8^, 1 × 10^9^, and 2 × 10^9^ CFU/mL) and phage with different MOI (0.01, 0.0001, 0.000001, and 0.00000001 at each bacterial inoculum concentration). At each time point (0, 1, 3, 5, 7, 9, 12, and 24 h), the bacterial growth and phage amplification were calculated as mentioned above.

### Effect of growth medium source on phage amplification

To select the optimal nutritional sources, we adapted the one-factor-at-a-time method, which is used in conventional scale-up processes. This approach is conducted by replacing one nutritional factor in the basal medium, while all the other factors are kept constant. In the experiments for the optimization of the carbon source, basal medium was supplemented with 0.5% (w/v) of glucose, sucrose, fructose, glycerol, and galactose, individually. To assess the effect of nitrogen sources, LB was supplemented with 0.1% (w/w) of casamino acid, peptone, gelatin, and glycine, individually. We also examined different surfactants (Tween 20, triton X-100, and SDS) at a concentration of 0.01% (v/v) to maximize the host strain surface for easy phage adherence. The influence of divalent cations on bacteriophage production was investigated by supplementing 0.01 M of calcium chloride or magnesium chloride to the LB medium.

### Experimental design for optimization of phage amplification

To determine the optimal levels of the selected variables, the experimental design was as per the central composite design method of RSM coded in Minitab (v16.2) software. Among the variables, glycerol, glycine, and calcium chloride were selected as the carbon, nitrogen, and ion source, respectively, as they displayed the highest phage production. The experimental setup consisted of 20 trials (Table 2), and all the experiments were conducted in triplicate. A second order polynomial equation was used for the analysis of phage production:

$${\text{Y}} = {\text{b}}\_0 + \sum {\text{b}}_{{\text{i}}} {\text{X}}_{{\text{i}}} + \sum {\text{b}}_{{{\text{ii}}}} {\text{X}}_{{\text{i}}}^{2} + \sum {\text{b}}_{{{\text{ij}}}} {\text{X}}_{{\text{i}}} {\text{X}}_{{\text{j}}} ,$$

where Y is the predicted response, b_0_ is the constant, b_i_ is the linear coefficient, b_ii_ is the quadratic coefficient, b_ij_ is the interaction coefficient, X_i_ is the independent variable, X_i_^2^ is the squared effect, and X_i_X_j_ is the interaction effect. The quadratic model was visualized as counterplots and the response surface curve was generated using Minitab (v16.2) for each variable. The correlation between μ and yield was analyzed using the regression model in the same software. Statistical analysis of the model was conducted using ANOVA and p < 0.05 was considered as significant.

## Supplementary Information

**Additional file 1.** Influence of the media supplement on the growth of host bacteria examined with carbon sources (**a**), nitrogen sources (**b**), divalent sources (**c**), and surfactants (**d**). The experiment was performed in triplicate.

## Data Availability

The datasets used and/or analysed during the current study are available from the corresponding author on reasonable request.

## Abbreviations

MOI: Multiplicity of infection
RSM: Response surface methodology
CCD: Central composite design
PFU: Plaque forming unit
OD: Optical density
LB: Luria–Bertani
ANOVA: Analysis of variance
