# Supplementary figures and images for: Strategy for mass production of lytic Staphylococcus aureus bacteriophage pSa-3: contribution of multiplicity of infection and response surface methodology
Source: Microb Cell Fact. 2021 Mar 2;20:56. doi: 10.1186/s12934-021-01549-8 (PMC7923500; doi:10.1186/s12934-021-01549-8)

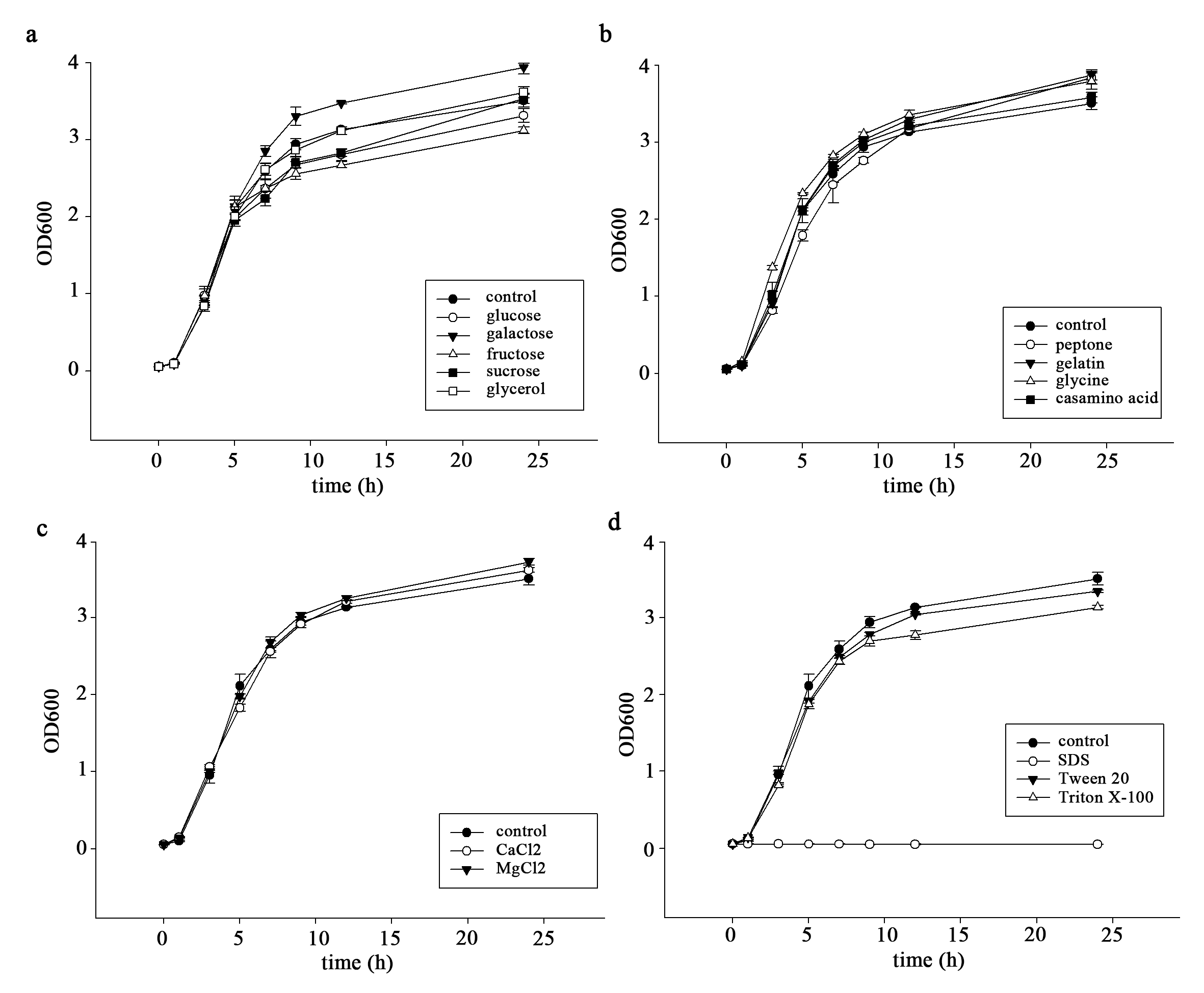

Supplement: Supplementary file 1 — Additional file 1. Influence of the media supplement on the growth of host bacteria examined with carbon sources (a), nitrogen sources (b), divalent sources (c), and surfactants (d). The experiment was performed in triplicate. [file 12934_2021_1549_MOESM1_ESM.tif]
